# Examining the impact of a universal social and emotional learning intervention (Passport) on internalising symptoms and other outcomes among children, compared to the usual school curriculum: study protocol for a school-based cluster randomised trial

**DOI:** 10.1186/s13063-023-07688-0

**Published:** 2023-11-02

**Authors:** Annie O’Brien, Suzanne Hamilton, Neil Humphrey, Pamela Qualter, Jan R. Boehnke, Joao Santos, Ola Demkowicz, Margarita Panayiotou, Alex Thompson, Jennifer Lau, Lauren Burke, Yizhuo Lu

**Affiliations:** 1 Manchester Institute of Education, The University of Manchester, Manchester, UK; 2 School of Health Sciences, University of Dundee, Dundee, UK; 3 The Manchester Centre for Health Economics, The University of Manchester, Manchester, UK; 4 Youth Resilience Unit, Wolfson Institute of Population Health, Queen Mary, University of London, London, UK

**Keywords:** Social and emotional learning, School-based, Universal intervention, Mental health, Well-being, Internalising symptoms, Children, Young people, Cluster randomised control trial

## Abstract

**Background:**

School-based universal social and emotional learning (SEL) interventions implemented during the transition to adolescence may be efficacious in preventing the development of mental health difficulties. This protocol describes a two-arm parallel cluster randomised controlled trial to investigate the impact of a universal SEL intervention (*Passport,* compared to usual provision) on internalising symptoms (primary outcome), emotion regulation, well-being, loneliness, social support, bullying, academic attainment, and health-related quality of life in English primary school pupils aged 9–11 years. A developer-led trial demonstrated the feasibility, acceptability, and utility of *Passport*; this will be the first independent trial.

**Methods:**

Sixty primary schools will be recruited across the Greater Manchester city region and surrounding areas, involving 2400 pupils aged 8–9 at baseline. Schools will be allocated to the intervention arm to implement *Passport* over 18 weekly sessions or to the control arm to implement the usual school curriculum. Random allocation will be at school level following completion of baseline measures, with minimisation to ensure balance across trial arms in school size and free school meal eligibility. Measures will be collected at baseline, post-intervention (12 months post-baseline), and at 12 months follow-up (24 months post-baseline). The primary outcome analysis (intervention effects on internalising symptoms at post-intervention) will comprise a two-level (school, child) hierarchical linear model, following the intention-to-treat principle. Additional analyses will be undertaken to assess intervention effects on secondary outcomes, maintenance effects for all outcomes, intervention compliance moderator effects, subgroup moderator effects, and mechanisms underpinning intervention effects on the primary outcome. A mixed-methods implementation and process evaluation will examine factors that influence implementation, and a health economic evaluation will assess the cost-effectiveness of the intervention.

**Discussion:**

Findings will provide educators with crucial knowledge of whether and how increasing emotion regulation through a universal intervention impacts internalising symptoms and a range of related outcomes. Findings will also inform policy related to the promotion of mental health among children and young people. If the intervention is found to be efficacious in reducing internalising symptoms and is also cost-effective, it may offer high potential as a preventative intervention for widespread implementation.

**Trial registration:**

ISRCTN12875599; registered on 24 November 2022

**Supplementary Information:**

The online version contains supplementary material available at 10.1186/s13063-023-07688-0.

## Introduction

### Background and rationale {6a}

Current data indicates that 16% of 5–16-year-olds experience mental health difficulties (MHDs). These difficulties, defined as enduring, maladaptive changes in thoughts, feelings, and/or behaviour, impair quality of life and are concurrently and prospectively associated with academic attainment and other salient outcomes [1]. Children with MHDs are more likely to experience social difficulties later in childhood, higher rates of mental health difficulties in adolescence, and perform poorer in exams at age 16 years compared to their same aged peers [2]. The transition from childhood to adolescence (between 9–11 years) appears to be crucial, given the occurrence of major physical, psychological, and social changes in this period [3]. Half of all lifetime mental disorders emerge by age 14 years and 75% by age 24 years [4], and there is a clear increase in the prevalence of mental health difficulties from childhood to adolescence [5]. Common MHDs that emerge in this developmental phase include an increase in internalising symptoms, characterised by a disturbance in mood or emotion, such as depression and anxiety [6, 7]. In particular, there is a marked increase in internalising symptoms among girls [5, 8]. Loneliness is also highly prevalent in early adolescence, with data suggesting that loneliness in middle childhood precedes depression in adolescence [9] and can predict other later MHDs [10].

#### Universal school-based prevention programmes: the role of social and emotional learning in alleviating internalising symptoms

Improving understanding of the role that schools can play in promoting mental health and reducing loneliness and related MHDs is a current national [11] and global [12] public health priority. Their wide reach, prolonged period of engagement, and central role in communities make schools ideal settings in which to implement universal interventions to prevent the development, maintenance, or escalation of MHDs among children and young people (CYP) [13].

Universal school-based interventions target all children regardless of level of difficulties or risk and align with the public health approach to mental health promotion within the UK. They are potentially more cost-effective than targeted/indicated approaches, may serve to reduce stigma, and, critically, can influence outcomes for CYP who would not otherwise access the support they need through usual care pathways [14]. Such an approach also aligns with the UK Government’s Loneliness Strategy, with a focus on creating connected (school) communities [15]. Universal school-based social and emotional learning (SEL) interventions aim to develop the social and emotional skills of all CYP through explicit instruction in the context of learning environments that are safe, caring, well-managed, and participatory [14]. The Collaborative for Academic, Social and Emotional Learning (CASEL) defines social-emotional competence in terms of five broad and interrelated skills: self-awareness, self-management, social awareness, relationship skills, and responsible decision-making [16]. Within the domain of self-management, emotion regulation is particularly important in preparing young people to cope with a wide range of stressors and challenges in daily life [17]. Crucially, as learning is a social process, it makes sense that the range of SEL skills can also support academic success (via, for example, improving engagement in the classroom) [14]. In the longer term, studies highlight the predictive utility of childhood social and emotional competencies for mental and social health and labour market outcomes in later life [18].

Meta-analytic evidence demonstrates the effectiveness of universal, school based SEL interventions in producing meaningful improvements in a range of salient outcomes related to mental health and well-being [19]. They have been shown to reduce internalising symptoms (effect size (ES) d/g = 0.19–0.24) [17, 19], with emergent evidence of sustained effects [20]. Furthermore, there is tentative evidence that they can reduce loneliness [21]. Because data suggests that children with internalising symptoms often show poorer social skills and emotional regulation capacities, acquiring such skills early has considerable utility, especially at a time when they are emerging and not yet trait-like or habitual. A universal intervention implemented as children become adolescents may be particularly beneficial to reduce many lifetime cases of MHDs that begin during adolescence [7].

#### Passport

Passport is a universal SEL intervention for 9–11-year-olds. The principal aim of the programme is to increase children’s abilities to cope with everyday difficulties by developing positive coping strategies. Each session is based around a comic strip that follows the adventures of two children and a friendly dragon. Children develop their own positive strategies to deal with problems through engaging activities: reading the comic strips, discussion, role play, and games [22]. Passport exemplifies the principles of SEL interventions by helping children to learn, expand, and consolidate their repertoire of social and emotional skills, enabling them to navigate more specific interpersonal difficulties such as bullying and stressful daily challenges.

Passport has a theoretical basis in coping theory [22] and aims to nurture coping flexibility by teaching a range of coping strategies. Coping flexibility involves the abilities to monitor and evaluate coping strategies, discontinue an ineffective strategy, and implement an alternative strategy that meets situational demands [23, 24]. Coping flexibility is associated with fewer mental health difficulties, particularly internalising symptoms [23, 24]. It is hypothesised to manifest through the initiation of more effective emotion regulation (defined as the processes involved in modifying the intensity, quality, duration, speed of elicitation, and recovery of emotional states in service of adaptation in situations that trigger unwanted feelings [25]). The negative cycle between difficulties with emotional regulation and the increase in unwanted feelings that is proposed to underpin the development of internalising symptoms [24] is therefore disrupted before it becomes consolidated and habitual. Delivering Passport *during* the transition from childhood to adolescence may therefore be a developmentally optimal strategy for nurturing resilience across the lifespan since it targets children just prior to the stage in which most MHDs emerge [4]. An association between emotion regulation and internalising symptoms in the transition to adolescence has been established empirically [26, 27] but it has not been established whether it is amenable to intervention.

There is an emerging evidence base for Passport*.* A small, developer-led trial in Canada demonstrated the feasibility (e.g., implemented as planned), acceptability (e.g., children enjoyed the comic strip format, high level of teacher appreciation of training in delivering intervention), and utility (e.g., improved coping and social skills) of the intervention, providing preliminary qualitative and quantitative evidence for the perceived mechanisms of a greater repertoire of coping strategies [28]. However, there is no independent evidence of Passport’s efficacy. In England, it is currently being phased into the education system by the Partnership for Children (and implemented in 115 schools to date). A robust, independent trial is, therefore, extremely timely.

While the SEL evidence base is well-advanced with respect to the basic question of ‘what works’, there is much still to learn, particularly in relation to: how and why interventions work (change mechanisms underpinning outcomes and implementation moderator effects) [19, 29]; for whom interventions work best (subgroup moderator effects) [13, 19] when interventions work (timing of effects, including both maintenance and sleeper effects) [13, 19]; the range of outcomes for which interventions work (scope) [13]; and at what cost they work [30]. Also required is further understanding of the cultural transferability of programmes and whether interventions can be effective when transported outside of their culture of development. Additionally, there is a need to extend the SEL evidence base beyond interventions implemented in the initial years of the primary school phase, from which much of the existing evidence is drawn [19].

### Objectives {7}

The trial’s objectives are as follows:

1. To examine the impact of *Passport* on children’s internalising symptoms at post-intervention in schools implementing the intervention compared to those implementing the usual school curriculum (primary outcome).
2. To determine the impact of *Passport* on a range of related outcomes, namely, emotional regulation, well-being, loneliness, bullying, peer support, and academic attainment (secondary outcomes), post-intervention in schools implementing the intervention compared to those implementing the usual school curriculum. Academic attainment will be assessed at 12-month post-intervention follow-up only (i.e., 24 months post-baseline).
3. To establish whether any intervention effects for primary and secondary outcomes are sustained at 12-month post-intervention follow-up (or emerge at 12-month follow-up, in the case of null impact at post-intervention).
4. To ascertain the cost-effectiveness of *Passport*.
5. To examine whether intervention effects vary by levels of implementation (specifically, dosage).
6. To examine whether primary intervention effects are mediated by changes in emotional regulation.
7. To determine whether low emotional regulation skills at baseline moderate primary intervention effects.
8. To investigate whether *Passport* is implemented as intended by the developer and what factors influence implementation.
9. To explore the perceptions and experiences of school staff and children in delivering and engaging with *Passport*.

### Trial design {8}

A two-arm (intervention vs. control) parallel cluster randomised controlled trial design, incorporating a mixed-methods implementation and process evaluation (IPE), will be used. Schools will be the unit of randomisation. Random allocation will take place following completion of baseline measures (T0), with minimisation ensuring balance across trial arms in school size and the proportion of children eligible for free school meals (FSM). This trial will follow a superiority framework (i.e., we hypothesise that the intervention is superior to usual practice).

Schools allocated to the intervention arm will implement *Passport* to Year 5 classes in the academic year 23/24. Implementing teachers will receive initial training followed by a booster session during the school term. Schools allocated to the control arm will continue to deliver the usual school curriculum. Child-level outcomes will be assessed annually at baseline (T0), post-intervention (T1), and 12-month post-intervention follow-up (T2).

The IPE will include a qualitative strand comprising case studies of 5 purposively sampled intervention schools (to include interviews with teaching staff and members of the senior leadership team alongside pupil focus groups), and a quantitative strand comprising teacher surveys focusing on usual practice in promoting SEL, outcomes (such as stress), and implementation (among those in intervention schools).

### Patient and Public Involvement (PPI)

PPI will be led by our colleagues at Common Room, with representatives sitting in our wider project team, and input from their young research advisors (YRA), who are young people with an interest in supporting research around children and young people’s mental health and well-being, particularly around the influence of social media and school support. Collaboration with Common Room YRA enables this research to benefit from young people’s participation and involvement across the trial. Public involvement in research has been associated with improvements in research design and delivery, recruitment strategies and materials, and data collection tools [31]. Prior to data collection, Common Room YRA will meet with the research team, providing valuable perspectives on the process, presentation, and evaluation of qualitative and quantitative data generation approaches (e.g., look and feel of online surveys), and how key processes are experienced and consented to (e.g., ensuring that the data burden of the survey is acceptable). YRA are helpful in effectively gathering information and insight from target groups of other young people to make sure the supporting processes and relevant information are accessible and meaningful (e.g., ensuring that participant information and assent process is accessible to CYP). Common Room representatives will also provide support with creative ways of disseminating our findings to improve impact and consulting with other stakeholder groups (e.g., teachers) as appropriate. They provided input in meetings that focused on the development and final review of this protocol.

## Methods: participants, interventions, and outcomes

### Study setting {9}

The trial will take place across mainstream primary schools across the Greater Manchester city region and surrounding areas. The trial recruitment areas provide diversity in terms of urbanicity, socio-economic status, ethnicity, and other factors that will help to ensure that our research setting reflects the heterogeneity similar to that of England.

### Eligibility criteria {10}

Schools are eligible to participate in the trial if they have the following characteristics:

1. A mainstream primary school.
2. A non-independent school.
3. Provide education to pupils in Year 4 through to Year 6.
4. Have never delivered *Passport.*
5. In the Greater Manchester city region and surrounding areas.

Children are eligible to participate in the trial if the following are in place:

1. They attend a participating school.
2. They are in Year 4 in the 2022/2023 academic year (i.e., at T0).
3. Their parents/carers do not opt them out of the study.
4. They assent to participate.

Schools are not eligible to take part in the trial if they meet the following criteria:

1. Are non-mainstream, and/or independent schools.
2. Do not provide education to pupils in Year 4 through to Year 6.
3. Are delivering, or have previously delivered, the Passport intervention.
4. Are outside the trial recruitment area.

Children are not eligible to participate if the following are true:

1. They do not attend a participating school.
2. Their parents/carers opt them out of the study.
3. They do not assent to participate in the study.

### Who will take informed consent? {26a}

#### Quantitative data

The trial manager is responsible for obtaining a signed Memorandum of Agreement and a Data Sharing Agreement from participating schools before participant recruitment begins. All participating schools will then send information sheets and consent forms to parents/carers of Year 4 pupils prior to T0, providing them the opportunity to opt their child out of the study. Children will provide assent to participate in the study prior to completion of each annual wave of outcome data collection. Those whose parents/carers opt them out, or those who do not assent, will not take part in the study. However, in line with schools’ *in loco parentis* responsibilities, children opted out of the study in intervention arm schools will still receive the Passport intervention, which will become part of their Relationships and Health Education provision.

#### Qualitative data

The qualitative strand of the trial IPE will take place in the 2023/2024 academic school year. Staff delivering Passport and senior leadership team members in 5 purposively sampled intervention schools will receive an information sheet and consent form inviting them to take part in individual interviews. To participate, they must return a signed consent form. At least 3 weeks before our first scheduled visit, we will send a pack to schools to be sent home with each child with invitations to participate in a focus group. Each pack will include a child-friendly information sheet, as well as a parent information sheet, parent consent form, and demographic sheet to complete.

Parents will be asked to return their consent form to the school before our visit, and we will collect and confirm these on the day before meeting with children. At the start of the focus group, we will go through the children’s information sheet with them again to ensure understanding and provide the opportunity to ask questions, before asking them for written assent.

### Additional consent provisions for collection and use of participant data and biological specimens {26b}

N/A; no samples collected.

## Interventions

### Explanation for the choice of comparators {6b}

Schools allocated to the control arm of the trial will continue to provide the usual school curriculum from T0 to T2. This will include a range of approaches to promoting SEL. Based on our recent survey of over 600 primary schools [32], we can expect: (i) most staff to have received some training in SEL; (ii) SEL to be among schools’ top priorities; (iii) most schools devote curriculum time to SEL; and (iv) the ‘Social and Emotional Aspects of Learning’ programme [33] to be the default programme implemented. We will use surveys at T0 and T1 with teachers in participating schools to capture usual practice in promoting SEL in schools in the control arm (and also in the intervention arm to capture baseline provision and additional provision parallel to the intervention period).

### Intervention description {11a}

Passport is a universal SEL intervention designed and developed by Professor Brian Mishara at the University of Quebec. It was designed in collaboration with students, teachers, and parents [22] and brought to the UK by Partnership for Children.

Passport will be taught to Year 5 classes during the 2023/24 school year by teachers trained in the programme. It comprises five modules, delivered over 18 weekly sessions, which cover the topics of emotions; relationships; difficult situations; fairness, justice and what is right; and change and loss. The developmentally sequenced lessons are 50 min in length and follow the adventures of Olya and Milo, two children that discover a fantasy world with their friend Elly the dragon. The entertaining comic book format provides a foundation for activities in which children identify, experiment with, and evaluate the utility of different strategies for dealing with challenging situations [22]. Intervention materials (digital and hard copy) include detailed lesson plans, home activities, posters, comic strips, individual *Passport* booklets for each child, emotion flashcards, and participation certificates. The programme design encourages adaptation and personalisation, in line with the session goals, to meet individual classroom needs [22, 28].

### Criteria for discontinuing or modifying allocated interventions {11b}

Participants can withdraw from the study (i.e., do not complete outcome surveys and/or withdraw previously completed surveys) up to the point of anonymisation. Schools will be asked to report any adverse events, and these will be monitored by the trial’s data monitoring and ethics committee (see {5d}). Given the nature of the intervention, we do not anticipate adverse events related to its delivery that will lead to termination of the trial. As such, there are no criteria in place for trial termination or for modifying intervention allocation.

### Strategies to improve adherence to interventions {11c}

Teachers responsible for the delivery of Passport will receive 1 day of training and a booster session mid-implementation with Partnership for Children.

### Relevant concomitant care permitted or prohibited during the trial {11d}

Delivery of the usual SEL curriculum is permitted in intervention and control schools as detailed in {6b}.

### Provisions for post-trial care {30}

N/A; no risk of harm anticipated; no provisions prepared.

### Outcomes {12}

The primary outcome is internalising symptoms operationalised by the mean score of the KIDSCREEN-52 Moods and Feelings subscale [34] at T1.

Secondary outcome measures are as follows:

1. Emotional regulation as defined by mean scores on the Children Worry Management Scale [35] at T1 and T2.
2. Well-being defined as change in mean scores on the KIDSCREEN-52 psychological well-being subscale [34] at T1 and T2.
3. Loneliness defined by a numerical category via the three-item UCLA-Loneliness scale adapted for children [36] as recommended by the Office of National Statistics at T1 and T2.
4. Bullying defined by mean scores on the KIDSCREEN-52 bullying subscale [34] at T1 and T2.
5. Peer support defined by mean scores on the KIDSCREEN-27 peer and social support subscale [37] at T1 and T2.
6. Academic attainment measured by Standard Assessment Test mean scores at T2.
7. Health-related quality of life, defined as mean change in scores on the Child Health Utility 9D [38] measure from T0 to T1 (for use in cost-effectiveness analysis).
8. Internalising symptoms operationalised by the mean score of the KIDSCREEN-52 Moods and Feelings subscale [34] at T2.

### Participant timeline {13}

Figures 1 and 2

**Fig. 1:**
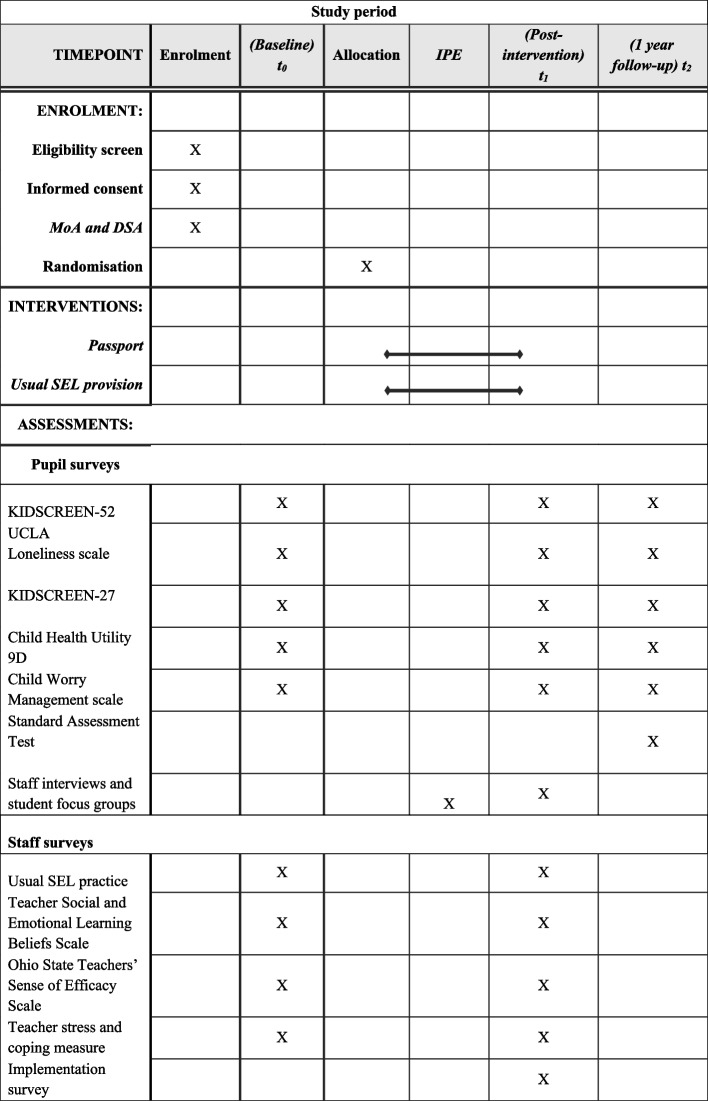
Data acquisition across trial timeline

**Fig. 2:**
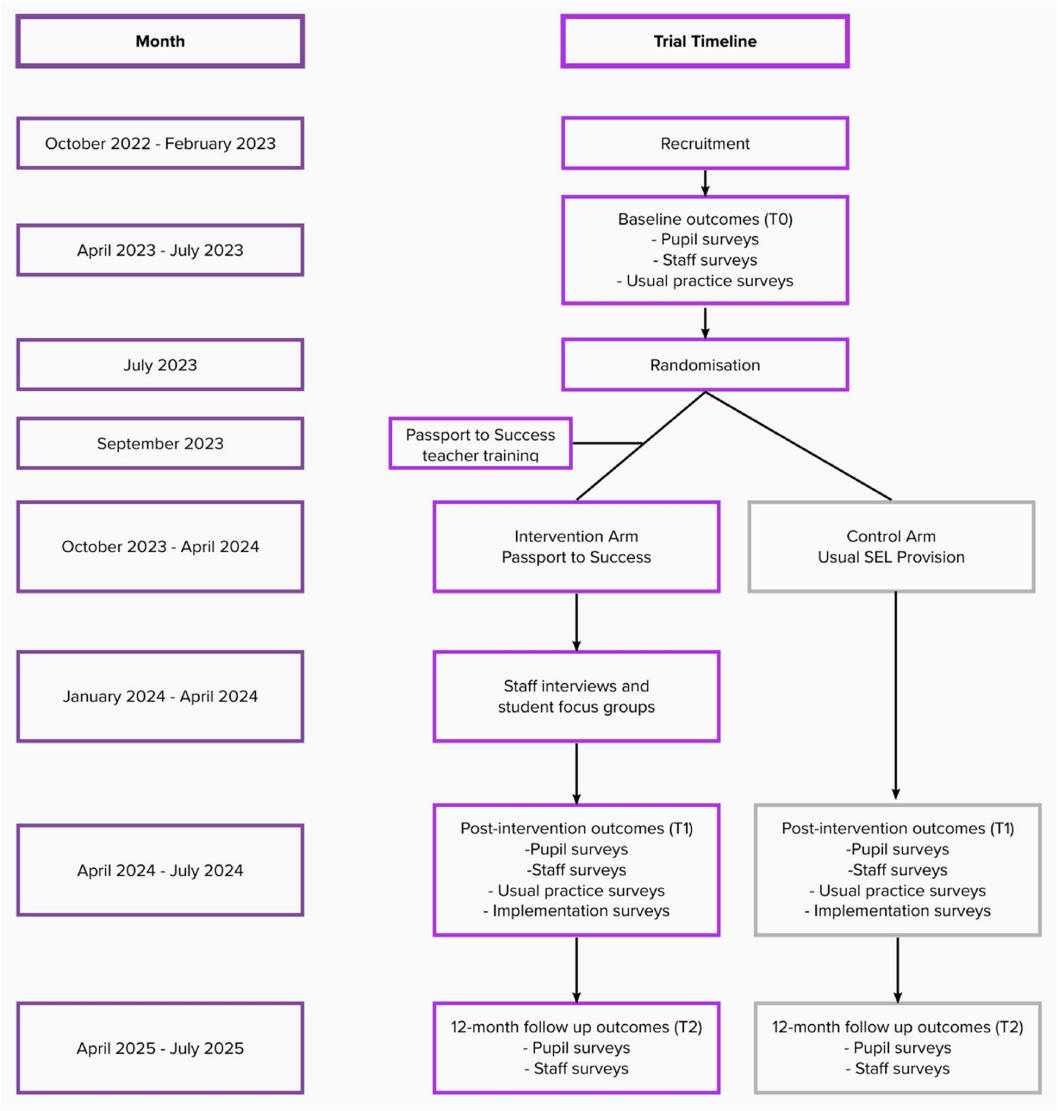
Flow diagram of trial timeline

#### Sample size {14}

Sample size calculations for the trial balanced considerations of what size of intervention effect might be considered important or meaningful for our primary outcome (internalising symptoms) in a public health context [13, 39], meta-analytic evidence indicating that SEL interventions typically reduce internalising symptoms by *d* = 0.19–0.24 [17, 19] and, pragmatic factors (i.e., the number of schools that could feasibly be recruited given funding and time constraints).

The following sample size calculations were carried out by the trial statistician in R, following the method outlined in Bloom, Richburg-Hayes and Black [40]. We conservatively power our primary outcome for a minimum detectable effect size (MDES) of 0.19. With alpha = 0.05, power = 0.80, intra-cluster correlation coefficient = 0.04 [41], 20% of outcome variance predicted by child-level covariates (including baseline scores; no variance explained by school-level covariates) [32], and with approximately 40 children per cluster [42] minimum of 52 schools are needed to detect an MDES of 0.16 (as well as to allow for an effective minimisation procedure and variation in underlying empirical parameters), with approximately 26 schools per arm and a total sample of 2080 children. We will oversample by c.15% to mitigate against school-level attrition. Our initial recruitment target is therefore 60 schools, yielding a total sample of 2400 children. However, we will recruit more schools, if possible, in order to maximise statistical power and provide greater protection against attrition.

For the qualitative strand, we are engaging 5 case study schools. In each of these schools, we will engage with the classroom teachers responsible for delivering Passport (*n* = 5) and members of senior leadership (*n* = 5). We will also engage with small groups of 5–6 children in each school, but will over-recruit to account for nonresponse and nonattendance, inviting 8 children to take part in each school, amounting to a possible n of 40 children in total. Using 5 schools allows us to explore a sufficient level of richness and detail of implementation in different contexts given our revisiting to the same schools over time.

### Recruitment {15}

Recruitment of schools began in October 2022 and will be completed in February 2023. The recruitment strategy follows successful practices established in our previous large-scale school-based projects, namely: relevant contacts in each LA (Local Authority) (e.g., primary education mental health lead) nominating interested schools to participate in the trial, followed by formal invitation from the trial manager. In parallel, we are also leveraging recruitment efforts through mutual stakeholders with strong existing relationships with primary schools in the city region and neighbouring areas, in addition to directly contacting schools, as necessary.

Following successful recruitment at school-level, we will commence recruitment of the trial’s target population: children in Year 4 at baseline (T0). All schools will send information sheets and consent forms to parents/carers of these children prior to T0, providing them the opportunity to opt their child out of the trial. Children will provide assent to participate prior to completion of each annual wave of outcome data collection. Those who do not assent will not complete outcome measures.

### Recruitment for qualitative IPE strand

We anticipate sampling schools by first requesting expression of interest from intervention schools to maximise the likelihood of engagement with qualitative data generation. To ensure maximum variability in contextual/compositional features (e.g., size, socio-economic status of catchment area) and usual SEL practice based on our baseline survey, 5 schools will be purposively sampled from those who express interest in being a case study site.

Staff (i.e., teachers and members of senior leadership) and children in the 5 purposively sampled intervention schools will be invited to take part in qualitative data generation for our IPE. Information sheets and consent forms will be sent to staff. Children will be invited to take part in focus groups by way of information packs. These will be sent to the schools in advance and given to each child to take home. Parents will be asked to return their consent form to the school and each child’s written assent will be sought prior to the commencement of the focus group. The interviews and focus groups will first take place during implementation of the intervention and at a second time point shortly after intervention delivery is completed.

## Assignment of interventions: allocation

### Sequence generation {16a}

Schools will be allocated 1:1 to either Passport or standard curriculum via minimisation; the allocation sequence will be generated via MinimPy software [43].

### Concealment mechanism {16b}

Schools will be allocated using MinimPy [43]. Allocation concealment will be ensured, as the allocation outcome will not be revealed until the school has been recruited into the trial and after all baseline measurements have been completed.

### Implementation {16c}

The independent statistician will generate the allocation sequence and allocate schools to the trial arms. We will minimise for percentage of free school meals (%FSM) and school size.

## Assignment of interventions: blinding

### Who will be blinded {17a}

Masking of allocation will apply only to the trial’s lead data analyst who is responsible for primary intent-to-treat analysis. Schools, trial participants, and the core research team will be masked to allocation until after recruitment and baseline measures are complete.

### Procedure for unblinding if needed {17b}

An unblinding procedure is not required as masking does not take place apart from for the lead data analyst, and there are no anticipated circumstances that would require them to be unblinded.

## Data collection and management

### Plans for assessment and collection of outcomes {18a}

Children’s names, unique pupil number (UPN), sex, free school meal eligibility, ethnicity, language, and special educational needs (SEN) provision status will be acquired directly from school records. Names and UPNs will be used to populate pupil survey lists for schools and to facilitate later data linkage (i.e., to academic attainment data held in the National Pupil Database). The other variables noted above will be used as co-variates in our statistical models and/or to provide a detailed description of the trial sample composition.

Quantitative data gathered from staff and pupil surveys will be collected via the secure, online survey platform Qualtrics. Each primary and secondary pupil outcome measure (see Table 1) was selected based on its brevity, age-appropriateness, psychometric properties, and feedback (e.g., accessibility, time burden) from YRA through our collaboration with Common Room (see {5d}). The sequence of measures in each participant survey will be randomised across participants and time points to reduce order bias. Academic attainment is measured using the Standard Assessment Tests collected from the National Pupil Database.

**Table 1:** Student outcome measures

Primary outcome	Measure	Subscale	Pearson’s correlation (*ρ*)^a^	Cronbach’s *α*	Age appropriateness of measure
Internalising symptoms	KIDSCREEN-52 [34]	Moods and emotions	0.52–0.59 [34]	0.86 [34]	Validated for 8–18-year-olds [34]
Secondary outcomes					
Emotional regulation	Children’s Worry Management Scale [35]	Coping	0.36–0.39	0.69 [35]	Validated for 6–12-year-olds [35]
Well-being	KIDSCREEN-52 [34]	Psychological well-being	0.57–0.60 [34]	0.89 [34]	Validated for 8–18-year-olds [34]
Loneliness	The three-item UCLA Loneliness scale adapted for children [36]	N/A	N/A	0.87–0.89 [44, 45]	Tested qualitatively with 10–15-year-olds [36]
Bullying	KIDSCREEN-52 [34]	Social acceptance	0.29–0.32 [34]	0.77 [34]	Validated for 8–18-year-olds [34]
Peer support	KIDSCREEN-27 [37]	Social support	0.36–0.39 [37]	0.94 [37]	Validated for 8–18-year-olds [37]
Health-related quality of life	Child Health Utilities 9D [38]	N/A	0.42–0.48 [38]	N/A	Validated for 7–11-year-olds [38]
Academic attainment	Standard assessment tests	N/A	N/A	N/A	N/A

^a^These numbers represent the convergent validity across instruments measuring similar constructs

We will undertake a comprehensive, mixed methods implementation and process evaluation. First, teachers in participating schools will complete a ‘usual SEL practice’ survey adapted from our previous research [46] at baseline (T0) and at the end of the first academic year (T1) to establish the counterfactual to Passport in control schools and the level of programme differentiation in intervention schools.

Second, teacher- and classroom-level outcome measures administered at T0 and T1 will capture teachers’ perception of SEL culture [47], stress, and self-efficacy in classroom management [48]. The following measures will be used: the Teacher Social and Emotional Learning Beliefs Scale [49], the brief teacher stress and coping measure [50], and the Ohio State Teachers’ Sense of Efficacy Scale classroom management subscale [51]. At post-intervention (T1), teachers in intervention schools will also complete an implementation survey based upon our previous work [29]. The survey captures dosage, adherence, quality, responsiveness, and reach of the intervention. Third, qualitative data from staff interviews (with both implementing teachers and members of the senior leadership team) and pupil focus groups in intervention schools will facilitate further exploration of implementation factors and nuanced considerations, such as adaptations and reasoning for these, programme acceptability, and student reach and responsiveness, while also highlighting factors affecting implementation. Qualitative data will be first generated during implementation of the intervention, through interviews with class teachers and focus groups with school children. At a second timepoint shortly after intervention delivery is completed, further interviews with class teachers, staff from the senior leadership team (SLT) and focus groups with children will be conducted. Data generation will be guided by interview and focus group schedules, reviewed and developed in collaboration with YRAs at Common Room.

Draft data collection documents can be found in the supplementary materials. Standard processes to promote data quality will be undertaken, including for example valid value and range checks. In addition, data cleaning processes will be mirrored for a minimum of 5% of the dataset by a third party (i.e., someone in the project team other than the trial/data manager, JS) to ensure replicability. A Data Management Plan has been created and is publicly available at https://dmponline.dcc.ac.uk/public_plans (Kavli2021-0000000019).

### Plans to promote participant retention and complete follow-up {18b}

To promote retention, all participating schools will receive feedback on outcomes across the trial generally and their school specifically. To guard against differential attrition, schools allocated to the control arm will receive Passport training and materials (if the alternative hypothesis is met) or the financial equivalent (if the null hypothesis is met).

To complete follow-up, the trial manager will send reminder emails and telephone calls (as needed) to teachers of classes with missing teacher or pupil data to maximise the volume of follow-up data collected.

### Data management {19}

The trial manager is responsible for cleaning and coding of study data with help from the team’s doctoral students. Survey data will be collected electronically and stored in an encrypted VeraCrypt container (AES-256 algorithm) located on the University of Manchester’s Research Data Storage Systems. Access to this data is limited to named users and requires dual factor authentication. Opt-out consent forms will be stored in a locked filing cabinet in the Ellen Wilkinson Building at the University of Manchester, with access for Passport team members only. Encrypted audio recorders will have their data uploaded to a secure setting as soon as possible and original data deleted from the device.

Identifying data (first name, surname, UPN) will be obtained from the schools for matching with attainment data and exported securely to the Office for National Statistics Secure Research Service (ONS SRS).

### Confidentiality {27}

We will collect the pupils' forename, surname, UPN, and socio-demographic data noted above from participating schools; this will be pseudonymised upon receipt, allowing participant data to be deleted if they withdraw from the trial before T1. Data will only be kept for as long as is necessary to meet project objectives, after which it will be destroyed. The project also entails opt-out parental/carer consent, meaning that opt-out forms will be received at various points in the project lifespan. All this information will be stored in accordance with the data management plan (see {19}).

The statistical modelling required to answer the project’s questions will require pseudonymised data. Once all analyses are completed, data will be fully anonymised. The required keys to transform pseudonymised data into identifiable data will be stored within the encrypted VeraCrypt drives, within UoM shared folders that require access clearance and dual factor authentication. No identifiable participant information will be reported in ​publications. The potential for internal re-identification of teachers by colleagues in case study schools will be made known to potential participants during recruitment for the qualitative strand.

### Plans for collection, laboratory evaluation, and storage of biological specimens for genetic or molecular analysis in this trial/future use {33}

N/A; no biological samples collected.

## Statistical methods

### Statistical methods for primary and secondary outcomes {20a}

In line with open science guidance [52], the authors aim to produce Registered Reports addressing study objectives, each of which will contain a detailed, prospective statistical analysis plan (SAP; or, the equivalent in the case of qualitative data). Following in principle acceptance of the Stage 1 submission of each Registered Report, these will be published and housed in the Open Science Framework (OSF) and the ISRCTN trial registry. For any study outputs that are not published as Registered Reports, we will pre-register analysis plans in the same repositories.

Here, we briefly outline our statistical methods for primary and secondary outcomes. Analyses will be undertaken using Mplus [53] and R [54]. Our primary outcome analysis will be undertaken by the masked lead analyst (JB). In brief, a 2-level random intercepts fixed slopes hierarchical linear model will be fitted for the primary outcome (level 1 = CYP; level 2 = schools). Internalising symptoms will be regressed on the treatment assignment alongside key CYP-level (baseline value in outcome) and school-level characteristics used in the minimisation procedure (%FSM, School Size). Children’s outcomes will be analysed per allocation, irrespective of their level of participation (i.e., intention-to-treat). Passport will be evaluated as potentially effective if the analysis shows a shows lower average internalising scores in the intervention condition and the bootstrap confidence interval for the coefficient does not include ‘0’.

The same analytic approach will be applied for ​​secondary outcomes at T1 (in place of internalising symptoms) with the T0 (baseline) outcome as a control variable to investigate potential effects at T2. As the trial is planned for a single primary endpoint (see above), these analyses of secondary outcomes and endpoints are exploratory in nature and will be described as such. Therefore, no correction for multiplicity is applied.

### Interim analyses {21b}

No interim analysis is planned.

### Methods for additional analyses {20b}

As noted above, analysis plans detailing approaches to quantitative and qualitative data analyses pertaining to the trial’s remaining objectives (e.g., detailing Complier Average Causal Effect (CACE) models for implementation moderator effects, multi-level mediation models for subgroup effects, health economic evaluation plan (HEAP) for cost-effectiveness evaluation) will be developed and submitted for external review and publication (i.e., as Registered Reports) prior to the conclusion of data collection.

All qualitative data will be analysed using reflexive thematic analysis [55] as follows: familiarisation with the data; systematic line-by-line coding; searching for themes across codes; reviewing and refining formative themes; defining and naming themes and developing a written report. Coding and theme development will be guided by a framework grounded in reviews and conceptual work on promotion and prevention programme implementation [56]. This process will be facilitated and organised using NVivo [57].

### Methods in analysis to handle protocol non-adherence and any statistical methods to handle missing data {20c}

The analysis plan (see {20b}) will detail our approach to handling missing data through robust methods, for example, the use of full information maximum likelihood (FIML) in our CACE analysis.

### Plans to give access to the full protocol, participant level-data and statistical code {31c}

The trial protocol, analysis plan, and HEAP will be pre-registered and published prior to commencement of data analysis. ​The anonymised quantitative and qualitative datasets will be deposited in the Open Science Framework.

## Oversight and monitoring

### Composition of the coordinating centre and trial steering committee {5d}

The study Principal Investigators will lead the project, with support from the Co-Investigators, ensuring it is delivered to time and budget. Core team meetings will take place on a weekly basis. Strands and work packages will be coordinated at these meetings so that work is tightly integrated and focused on addressing the overarching aims and objectives of the project, and that the various strands of work are brought together.

A Trial Steering Committee (TSC), which incorporates a data monitoring and ethics sub-committee (DMEC), will meet 3 times per year to monitor the progress of the project and support strategic decisions. The TSC and DMEC comprises a range of invited external stakeholders (e.g., independent academic chair, educators, Common Room, Education Trials Unit, Youth Sports Trust).

### Composition of the data monitoring committee, its role and reporting structure {21a}

The Data Monitoring and Ethics Committee (DMEC) operates as an independent advisory group. Its aim is to provide rigorous feedback on data-related and ethical issues. It consists of four members, each with different expertise. Two of its members are experienced statisticians, acting as quantitative advisors, with the remaining members acting as experts on safeguarding and social emotional learning. Meetings occur three times a year and are structured in a way that allows for the trial’s statistician to remain blinded. The DMEC has no competing interests.

### Adverse event reporting and harms {22}

A distress and safeguarding protocol, approved by the University of Manchester’s Research Ethics Committee (UREC; Ref: 2022-14050-24401), details a stepwise approach to reviewing and responding to a display of distress during pupil focus groups. Disclosure of safeguarding concerns is reported to the on-site safeguarding officer; should the safeguarding lead indicate that they do not intend to act on this information, and we disagree, we will consult with the Research Ethics Officer (as per the University of Manchester Standard Operating Procedure for Incident Responses). Any adverse events are reported to DMEC.

More broadly, the research team are cognizant of the possibility that a universal, school-based intervention has the potential to produce unintended negative consequences, leading some authors to recommend measurement, investigation and reporting of individual symptom deterioration as standard [58]. This follows a small number of studies reporting small iatrogenic effects for certain interventions, particularly for participants with elevated levels of mental health symptoms at baseline (including, for example, the UK-based MYRIAD trial focusing on mindfulness training [59]). In the case of this trial, we consider the risk of such effects to be minimal, given the nature of the Passport intervention compared to those for which negative effects have been documented [58] and the empirical distribution of intervention effect sizes for universal SEL interventions (which overwhelmingly favours positive intervention effects [60]). Indeed, in the aforementioned MYRIAD example, the content of Passport is arguably closer to the teaching as usual condition, which was documented in terms of the presence of Personal, Social and Health Education and SEL curricula [59]).

Nonetheless, our trial objectives—and the analyses pertaining to each—enable us to determine the presence of any manner of intervention effects (inclusive of positive, null, or negative/iatrogenic) at both ITT and subgroup level, and the planned use of registered reports means that any negative effects will be clearly documented. In addition, qualitative data generation undertaken in pursuit of objective 9 will enable us to document the perceptions of both staff and pupils regarding the impact of Passport (as above, whether positive, null, or negative/iatrogenic), and any proposed underpinning mechanisms.

### Frequency and plans for auditing trial conduct {23}

The TSC and DMEC have governance and oversight responsibilities for the trial and advising the research team as necessary. They will meet with the research team 3 times per year to monitor the progress of the project and support strategic and operational decisions.

### Plans for communicating important protocol amendments to relevant parties (e.g., trial participants, ethical committees) {25}

The trial sponsor, DMEC, TSC, and implementation sites will be informed of protocol amendments, and the published protocol updated, following discussion via team meeting(s).

### Dissemination plans {31a}

We will disseminate our research findings in consultation with the YRA via (i) a main project report published online; (ii) reports and videos for schools/LAs and CYP; (iii) National Elf Service (NES) package of dissemination activities including blogs/blog shots, social media, and other digital services, or equivalent; (iv) peer-reviewed publications and conference presentations; and (v) submission of programme information and evidence to the Early Intervention Foundation for assessment in relation to their Guidebook. Finally, participating schools will be provided with an anonymised feedback report describing survey results in tabular and data visualisation formats.

## Discussion

This will be the first independent trial of the effectiveness of *Passport*. A trial of this nature is needed given the increased prevalence of internalising symptoms, and the potential of universal, school-based SEL interventions to address such difficulties. More broadly, the trial will enhance our understanding of whether the association between emotional regulation and internalising symptoms is amenable to intervention in the transition from childhood to adolescence. This will provide educators with crucial knowledge of whether, and how, increasing emotion regulation through universal intervention impacts mental health and related outcomes. It is anticipated that the findings will inform the future design of school-based materials and policy relating to the promotion of mental health in educational settings.

Furthermore, the trial will go beyond ‘what works’ to also consider ‘how’ and ‘why’ interventions work through an examination of the mechanisms underpinning change, something highlighted as a priority area for future intervention research [19], but, as yet, remains largely unaddressed. Similarly, CACE estimation remains neglected in school-based intervention research [61], particularly in the context of cluster randomised trials, and despite its utility in rigorously demonstrating implementation moderator effects. Our planned CACE analysis will determine whether the intervention effects noted in ITT models are influenced by intervention compliance. The trial will also contribute to intervention research knowledge in another neglected area, by addressing the question of ‘for whom’ the intervention works, through a subgroup moderator analysis to identify any differential effects.

Finally, the trial will aim to determine whether *Passport* is cost-effective. This is crucial in this area of research: despite the proliferation of universal preventive approaches to mental health (including SEL) in education systems worldwide, there remains a dearth of rigorous health economic evaluations [62]. A cost-effectiveness analysis will help local and national policymakers determine whether to allocate scarce resources to interventions such as *Passport*.

## Trial status

This is protocol V.2 September 2023. School recruitment began in October 2022 with the signing of MOAs (Memorandum of Agreement) and DSAs (Data Sharing Agreements), running until February 2023. Student participant recruitment, defined as the first day of student data collection, is scheduled to begin in April 2023 and finish in July 2023.

### Supplementary Information

**Additional file 1. **Data collection tools.

**Additional file 2.** SPIRIT 2013 Checklist: Recommended items to address in a clinical trial protocol and related documents.

## Data Availability

An anonymized data set will be made publicly available through the Open Science Framework.

## Abbreviations

CASEL: The Collective for Academic, Social and Emotional Learning
CACE: Complier average causal effect
CYP: Children and young people
DSA: Data sharing agreement
FIML: Full information maximum likelihood
FSM: Free school meals
GM: Greater Manchester
HEAP: Health Economic Evaluation Plan
HLM: Hierarchical linear modelling
IPE: Implementation and process evaluation
ITT: Intention to treat
ISRCTN: International Standard Randomised Controlled Trial Number
LA: Local authority
MDES: Minimal detectable effect size
MHD: Mental health difficulty
MOA: Memorandum of Agreement
NES: National Elf Service
ONS SRS: Office for National Statistics Secure Research Service
OSF: Open Science Framework
QALY: Quality adjusted life years
RTC: Randomized controlled trial
SAP: Statistical analysis plan
SEL: Social and emotional learning
SEN: Special Educational Needs
UPN: Unique pupil number
UREC: University of Manchester’s Research Ethics Committee
YRA: Young Researcher Advisor
