# Correction: Examining the impact of a universal social and emotional learning intervention (Passport) on internalising symptoms and other outcomes among children, compared to the usual school curriculum: study protocol for a school-based cluster randomised trial

**DOI:** 10.1186/s13063-023-07895-9

**Published:** 2024-01-09

**Authors:** Annie O’Brien, Suzanne Hamilton, Neil Humphrey, Pamela Qualter, Jan R. Boehnke, Joao Santos, Ola Demkowicz, Margarita Panayiotou, Alex Thompson, Jennifer Lau, Lauren Burke, Yizhuo Lu

**Affiliations:** 1 Manchester Institute of Education, The University of Manchester, Manchester, UK; 2 School of Health Sciences, University of Dundee, Dundee, UK; 3 The Manchester Centre for Health Economics, The University of Manchester, Manchester, UK; 4 Youth Resilience Unit, Wolfson Institute of Population Health, University of London, London, Queen Mary UK

**Correction: Trials 24, 703 (2023)**

**https://doi.org/10.1186/s13063-023-07688-0**

Following publication of the original article [1], we have been notified that Line 366 of the manuscript states the “intra-cluster correlation coefficient = 0.04” and cites reference 38 (Stevens et al., 2012). This reference is incorrect. The reference should be the following paper:

Hayes D, Moore A, Stapley E, Humphrey N, Mansfield R, Santos J, Ashworth E, Patalay P, Bonin E, Evans-Lacko S, Moltrecht B. School-based intervention study examining approaches for well-being and mental health literacy of pupils in Year 9 in England: study protocol for a multischool, parallel group cluster randomised controlled trial (AWARE). BMJ open. 2019 Aug 1;9(8): e029044.

The original article has been corrected.
