# Supplementary material for: Examining the impact of a universal social and emotional learning intervention (Passport) on internalising symptoms and other outcomes among children, compared to the usual school curriculum: study protocol for a school-based cluster randomised trial
Source: Trials. 2023 Nov 2;24:703. doi: 10.1186/s13063-023-07688-0 (PMC10621084; doi:10.1186/s13063-023-07688-0)
Supplement: Supplementary file 1 — Additional file 1. Data collection tools. [file 13063_2023_7688_MOESM1_ESM.zip › Additional file 1. /Teacher interview schedule visit 1 version 2 .docx]

**
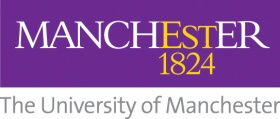
**

**Kavli Teacher Interview Schedule visit 1**

**Introduction**

**Preamble**

*Provide participant with copy of information sheet (they will have already received a copy in email), offer a summary of key areas, and confirm that they have read this and understood*

Our conversation will take about an hour. There are no right or wrong answers, we are just interested in what you think. This evaluation is separate from the Passport intervention team so we don’t have any vested interests in the intervention, and we want you to be honest with us about how you are finding Passport.

We will record our conversation so that we can write down all the things you tell us. We may write about the things that you tell us in reports, but other people outside of your school won’t be able to tell that it’s you. At the end of the project, we will share a written version of the conversations that we have together, but we won’t tell anyone your name and people who see this won’t be able to tell that it’s you. Because we are only visiting a small number of schools, it is possible that colleagues in the school may know that it is you in our report when we write about how teachers and children have experienced delivering Passport. However, we are not asking questions that we think would affect your reputation, and people outside of your school will not be able to tell that it is you.

**Ask**: are you happy to chat to me today about your experiences of delivering Passport with your class?

**Ask**: do you have any questions before we start?

*Provide consent form and take written consent from the participant before beginning.*

**Ask**: Is it okay for me to start recording now?

**Schedule**

*Various prompts shown in relation to questions. More widely, probe answers to help unpack points, and elicit details and concrete examples.*

**A. Teacher and school background**

*Looking for information about the teacher’s background and role in the school, and how they view wellbeing within their role and day-to-day classroom life*

1. Can you briefly describe for me your experience in education?
2. Can you briefly describe your role and position in this school?
3. How would you define social and emotional learning in schools?
   1. Can you give me an example of social and emotional learning?
   2. What do you think is useful about social and emotional learning in schools?
   3. Have you had any training before Passport about social and emotional learning or related areas of practice?
4. Can you tell us a little bit about your school?
   1. Can you tell us generally about the families and children that you work with as a school? How does this context affect the policy and practices in your school?
   2. What’s the ethos like in your school?
   3. Can you tell us a little bit about the social dynamics in your class at the moment? Do you think this reflects the dynamics in the school more generally? (Examples if needed: friendships and relationships, behaviour patterns)
   4. Can you tell us about the social and emotional needs of your class at the moment? Do you think this reflects the needs of the school more generally? (Examples if needed: difficulties with friendship, managing difficult feelings, coping with stress and loss) Do you think that links back to the communities you work with as a school?
5. Before you began delivering Passport, what kinds of things did you do to support children’s social and emotional development in your classroom?

**B. Preparing for Passport**

*Looking for information about initial impressions, teacher buy-in, the process of school involvement, training experiences, and programme differentiation.*

1. Can you describe how your school became involved in delivering Passport?
   1. What do you think the reasons were that your school decided to deliver Passport?
   2. What did you think when you learned you would be delivering Passport with your class? Did this change as you began to prepare to deliver Passport? *(initial impressions and teacher buy in)*
2. Can you tell me about how you found the training for Passport? *(training experiences)*
   1. Can you tell me about anything you found helpful about the training?
   2. Can you tell me about anything you found less helpful about the training, or anything you think could have been done differently or better?
   3. What expectations did you have of Passport *after* the training?
   4. What expectations did you have of the benefits it could have for your class, if any?
3. What do you think are the similarities and differences between Passport and other wellbeing initiatives or practices in which the school has been involved? *(programme differentiation)*

**C. Implementation of Passport**

*Looking for information about dosage, fidelity and adaptation, reach and responsiveness, quality, and factors affecting implementation.*

1. Can you tell me about your overall impressions of Passport so far?
2. Roughly how many sessions of Passport have you delivered so far?
   1. How often have you been delivering these?
   2. How have you found it fitting these into the week?
3. We know that teachers quite often make changes to social and emotional learning lessons – changing scenarios or wording, leaving some parts out, etcetera. These might be big changes or little changes, and it might be something that’s changed *every* session or it might be about one particular activity one week. Have you made any changes to the sessions when you have been delivering them?
   1. Follow up to probe their answer – e.g., how often did they do X, to what extent?
   2. *If yes*: can you walk me through the thinking behind those changes?
   3. *If yes:* How have these changes worked in practice?
   4. *If no:* Were there any changes you have thought about making?
4. How do you think your students have been responding to Passport sessions?
   1. Can you give me any examples of how students have been responding?
   2. How do they respond to the materials?
   3. How do they react to the scenarios?
5. If other teachers were to deliver Passport in their classrooms, do you think there’s anything they should know about how to do it well?
6. Is there anything about your school that you think is helpful or unhelpful when you are trying to incorporate Passport?

**D. Impact and helpful aspects of Passport**

*Looking for information about impact for children, including any differential impact*

1. Are there any modules or sessions that are particularly well suited to the needs of your class? [Remind that file is available]
   1. You mentioned earlier [insert relevant school context or class need as noted in Section A] - how do you think that Passport relates back to that so far?
2. Do you think any of the children in your class have benefitted from Passport so far?
   1. In what ways do you think they have benefited?
   2. If unclear: ~~is this the same for all children, or~~ do you think particular children have benefitted more or less than one another? Can you tell me about why you think that is?
   3. Can you give me any examples of ways that you think children have benefitted?
   4. Do you think there are any patterns in how you are seeing these benefits? For instance, in groups of children with particular needs or circumstances?
   5. *If no:* can you tell me your thoughts about why Passport may not have benefitted children in your class?
3. We know interventions like Passport often have lots of aspects to them, and some things might be seen as really helpful, almost like “key ingredients” - from your experience of delivering Passport, what do you think the key ingredients are in it?

**Conclusion**

That’s all my questions for you. Is there anything else you would like to add?

*Stop recording now.*

Thank you so much for talking with us, it’s been helpful to talk to you and understanding your experiences of delivering Passport. We’ll be coming back again after you have finished delivering Passport, to talk with you and children in your class once you have completed the programme.

To remind you, we’re going to write about the things we’ve been discussing in reports but other people outside of the school won’t be able to tell that it’s you in those reports. We will share a written version of our conversations with other people, but we won’t tell them that this is you.

Do you have any questions for us now that we’ve finished? If you think of any questions later, you can email us again.
