# Supplementary material for: Examining the impact of a universal social and emotional learning intervention (Passport) on internalising symptoms and other outcomes among children, compared to the usual school curriculum: study protocol for a school-based cluster randomised trial
Source: Trials. 2023 Nov 2;24:703. doi: 10.1186/s13063-023-07688-0 (PMC10621084; doi:10.1186/s13063-023-07688-0)
Supplement: Supplementary file 1 — Additional file 1. Data collection tools. [file 13063_2023_7688_MOESM1_ESM.zip › Additional file 1. /Focus Group Demographic FormR1.docx]

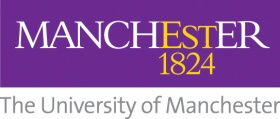


**Passport to Success**

**Information about your Child**

We will need the following information about the children taking part in this research. Having this information means we can better understand the different voices that are included in this project. If you are happy for your child to take part, please complete this form to tell us about your child and return it to their school along with the consent form.

**Your child’s full name:** ____________________________

**Age in years:** ____________________________

**Gender (please tick):**

- Girl
- Boy
- Other
- Prefer not to say

**Ethnicity:**

- White or White British
- Mixed or multiple ethnic groups
- Asian or Asian British
- Black, African, Caribbean, or Black British
- Another ethnic group
- Prefer not to say

**Is your child eligible for free school meals?**

- Yes
- No
- Prefer not to say

**Does your child have special educational needs and/or disabilities?**

- Yes
- No
- Prefer not to say

**Does your child speak English as an additional language?**

- Yes
- No
- Prefer not to say
