# Supplementary material for: Examining the impact of a universal social and emotional learning intervention (Passport) on internalising symptoms and other outcomes among children, compared to the usual school curriculum: study protocol for a school-based cluster randomised trial
Source: Trials. 2023 Nov 2;24:703. doi: 10.1186/s13063-023-07688-0 (PMC10621084; doi:10.1186/s13063-023-07688-0)
Supplement: Supplementary file 1 — Additional file 1. Data collection tools. [file 13063_2023_7688_MOESM1_ESM.zip › Additional file 1. /Child Outcome SurveyR1.docx]

**Passport to Success**

**Child self-report outcome measures**

**Internalising symptoms (*Mood and Emotions* subscale of KIDSCREEN-52)**

| Item | Response format | | | | |
| --- | --- | --- | --- | --- | --- |
| Instruction: Thinking about last week... | | | | | |
| Have you felt that you do everything badly? | Never | Seldom | Sometimes | Often | Always |
| Have you felt sad? | Never | Seldom | Sometimes | Often | Always |
| Have you felt so bad that you didn’t want to do anything? | Never | Seldom | Sometimes | Often | Always |
| Have you felt that everything in your life goes wrong? | Never | Seldom | Sometimes | Often | Always |
| Have you felt fed up? | Never | Seldom | Sometimes | Often | Always |
| Have you felt lonely? | Never | Seldom | Sometimes | Often | Always |
| Have you felt under pressure? | Never | Seldom | Sometimes | Often | Always |

**Emotion regulation (*Coping* subscale of the Children’s Worry Management Scale)**

| Item | Response format | | |
| --- | --- | --- | --- |
| I keep myself from losing control of my worried feelings | Hardly ever | Sometimes | Often |
| I talk to someone until I feel better when I am worried | Hardly ever | Sometimes | Often |
| I try to calmly settle the problem when I feel worried | Hardly ever | Sometimes | Often |

**Wellbeing (*Psychological wellbeing* subscale of KIDSCREEN-52)**

| Item | Response format | | | | |
| --- | --- | --- | --- | --- | --- |
| Instruction: Thinking about the last week… | | | | | |
| Has your life been enjoyable? | Not at all | Slightly | Moderately | Very | Extremely |
| Have you felt pleased that you are alive? | Not at all | Slightly | Moderately | Very | Extremely |
| Have you felt satisfied with your life? | Not at all | Slightly | Moderately | Very | Extremely |
| Have you been in a good mood? | Never | Seldom | Quite often | Very Often | Always |
| Have you felt cheerful? | Never | Seldom | Quite often | Very often | Always |
| Have you had fun? | Never | Seldom | Quite often | Very often | Always |

**Loneliness (UCLA 3-item loneliness scale)**

| Item | Response format | | |
| --- | --- | --- | --- |
| How often do you feel that you have no one to talk to? | Hardly Ever or Never | Some of the Time | Often |
| How often do you feel left out? | Hardly Ever or Never | Some of the Time | Often |
| How often do you feel alone? | Hardly Ever or Never | Some of the Time | Often |

**Bullying (*Social acceptance* subscale of KIDSCREEN-52)**

| Item | Response format | | | | |
| --- | --- | --- | --- | --- | --- |
| Instruction: Thinking about the last week… | | | | | |
| Have you been afraid of other girls and boys? | Never | Seldom | Quite often | Very Often | Always |
| Have other girls and boys made fun of you? | Never | Seldom | Quite often | Very often | Always |
| Have other girls and boys bullied you? | Never | Seldom | Quite often | Very often | Always |

**Peer support (Peer support subscale of KIDSCREEN-27)**

| Item | Response format | | | | |
| --- | --- | --- | --- | --- | --- |
| Instruction: Thinking about the last week… | | | | | |
| Have you spent time with your friends? | Never | Seldom | Quite often | Very Often | Always |
| Have you had fun with your friends? | Never | Seldom | Quite often | Very often | Always |
| Have you and your friends helped each other? | Never | Seldom | Quite often | Very often | Always |
| Have you been able to rely on your friends? | Never | Seldom | Quite often | Very often | Always |

**Health-related quality of life (Child Health Utilities 9D)**

| Item | Response format | | | | |
| --- | --- | --- | --- | --- | --- |
| Worried | I don’t feel worried today | I feel a little bit worried today | I feel a bit worried today | I feel quite worried today | I feel very worried today |
| Sad | I don’t feel sad today | I feel a little bit sad today | I feel a bit sad today | I feel quite sad today | I feel very sad today |
| Pain | I don’t have any pain today | I have a little bit of pain today | I have a bit of pain today | I have quite a lot of pain today | I have a lot of pain today |
| Tired | I don’t feel tired today | I feel a little bit tired today | I feel a bit tired today | I feel quite tired today | I feel very tired today |
| Annoyed | I don’t feel annoyed today | I feel a little bit annoyed today | I feel a bit annoyed today | I feel quite annoyed today | I feel very annoyed today |
| Schoolwork/homework | I have no problems with my schoolwork today | I have a few problems with my schoolwork today | I have some problems with my schoolwork today | I have many problems with my schoolwork today | I can’t do my schoolwork today |
| Sleep | Last night I had no problems sleeping | Last night I had a few problems sleeping | Last night I had some problems sleeping | Last night I had many problems sleeping | Last night I couldn’t sleep |
| Daily routine (things like eating, having a bath/shower, getting dressed | I have no problems with my daily routine today | I have a few problems with my daily routine today | I have some problems with my daily routine today | I have many problems with my daily routine today | I can’t do my daily routine today |
| Join in activities (things like playing out with your friends, doing sports) | I can join in with any activities today | I can join in with most activities today | I can join in with some activities today | I can join in with a few activities today | I can join in with no activities today |
