# Supplementary material for: Examining the impact of a universal social and emotional learning intervention (Passport) on internalising symptoms and other outcomes among children, compared to the usual school curriculum: study protocol for a school-based cluster randomised trial
Source: Trials. 2023 Nov 2;24:703. doi: 10.1186/s13063-023-07688-0 (PMC10621084; doi:10.1186/s13063-023-07688-0)
Supplement: Supplementary file 1 — Additional file 1. Data collection tools. [file 13063_2023_7688_MOESM1_ESM.zip › Additional file 1. /Teacher Outcome Survey and Usual SEL Practice Survey v.2R1.docx]

**Passport to Success**

**Teacher self-report outcome measures**

**Demographics**

| **Item** | **Response format** | | | | |
| --- | --- | --- | --- | --- | --- |
| Are you: | Male (including trans male) | Female (including trans female) | Non-binary | I describe myself in another way | Prefer not to say |

| **Item** | **Response format** |
| --- | --- |
| For how many years have you been a qualified teacher? | Drop-down menu or slider from 0-50 |

**Usual practice in social and emotional learning (original items based on lesson content of Passport curriculum)**

| **Item** | **Response format** | | | |
| --- | --- | --- | --- | --- |
| In a typical school year, please indicate the frequency with which you teach at least one lesson on the following topics with your class: | | | | |
| Valuing our differences and similarities | Never | Sometimes | Often | Always |
| Understanding and expressing our emotions | Never | Sometimes | Often | Always |
| Recognising other people’s feelings | Never | Sometimes | Often | Always |
| Helping each other and coping skills | Never | Sometimes | Often | Always |
| Friendship | Never | Sometimes | Often | Always |
| Challenges in friendship | Never | Sometimes | Often | Always |
| Dealing with frustration | Never | Sometimes | Often | Always |
| Dealing with stress | Never | Sometimes | Often | Always |
| Dealing with conflict | Never | Sometimes | Often | Always |
| Unfairness in daily life | Never | Sometimes | Often | Always |
| Dealing with bullying | Never | Sometimes | Often | Always |
| Unfairness and injustice in the world | Never | Sometimes | Often | Always |
| Coping with change | Never | Sometimes | Often | Always |
| Coping with loss | Never | Sometimes | Often | Always |
| Helping others in difficult situations | Never | Sometimes | Often | Always |

| **Item** | **Response format** | |
| --- | --- | --- |
| In a typical school year, do you teach social and emotional skills using a proprietary social and emotional learning curriculum? This includes programmes like Social and Emotional Aspects of Learning, Promoting Alternative Thinking Strategies, and FRIENDS. | No | Yes |

**Perception of social and emotional learning culture (Teacher Social and Emotional Learning Beliefs Scale)**

| **Item** | **Response format** | | | | |
| --- | --- | --- | --- | --- | --- |
| I feel confident in my ability to provide instruction on social and emotional learning | Strongly disagree | Disagree | Neither agree nor disagree | Agree | Strongly agree |
| I am comfortable providing instruction on social and emotional skills to my students | Strongly disagree | Disagree | Neither agree nor disagree | Agree | Strongly agree |
| Taking care of my students’ social and emotional needs comes naturally to me | Strongly disagree | Disagree | Neither agree nor disagree | Agree | Strongly agree |
| Informal lessons in social and emotional learning are part of my regular teaching practice | Strongly disagree | Disagree | Neither agree nor disagree | Agree | Strongly agree |
| I would like to attend a workshop to develop my students’ social and emotional skills | Strongly disagree | Disagree | Neither agree nor disagree | Agree | Strongly agree |
| I would like to attend a workshop to develop my own social and emotional skills | Strongly disagree | Disagree | Neither agree nor disagree | Agree | Strongly agree |
| I want to improve my ability to teach social and emotional skills to students | Strongly disagree | Disagree | Neither agree nor disagree | Agree | Strongly agree |
| All teachers should receive training on how to teach social and emotional skills to student | Strongly disagree | Disagree | Neither agree nor disagree | Agree | Strongly agree |
| My headteacher creates an environment that promotes social and emotional learning for our students | Strongly disagree | Disagree | Neither agree nor disagree | Agree | Strongly agree |
| The culture in my school supports the development of children’s social and emotional skills | Strongly disagree | Disagree | Neither agree nor disagree | Agree | Strongly agree |
| My headteacher does not encourage the teaching of social and emotional skills to students (reverse scored) | Strongly disagree | Disagree | Neither agree nor disagree | Agree | Strongly agree |
| My school expects teachers to address children’s social and emotional needs | Strongly disagree | Disagree | Neither agree nor disagree | Agree | Strongly agree |

**Self-efficacy in classroom management (Ohio State Teachers’ Sense of Efficacy Scale classroom management subscale)**

| **Item** | **Response format** | | | | | | | | |
| --- | --- | --- | --- | --- | --- | --- | --- | --- | --- |
| How much can you do to control disruptive behaviour in the classroom? | Not at all | - | Very little | - | Some influence | - | Quite a bit | - | A great deal |
| How much can you do to get children to behave in the classroom? | Not at all | - | Very little | - | Some influence | - | Quite a bit | - | A great deal |
| How much can you do to calm a student who is disruptive or noisy? | Not at all | - | Very little | -- | Some influence | - | Quite a bit | - | A great deal |
| How well can you establish a classroom management system with each group of students? | Not at all | - | Very little | - | Some influence | - | Quite a bit | - | A great deal |

**Perceived Stress**

| **Please tick the option that best applies:** | | | | | | | | | | | |
| --- | --- | --- | --- | --- | --- | --- | --- | --- | --- | --- | --- |
| **Item** | **Response format** | | | | | | | | | | |
| How stressful is your job? | Not stressful |  |  |  |  |  |  |  |  |  | Very stressful |

**Coping with Stress**

| **Please tick the option that best applies:** | | | | | | | | | | | |
| --- | --- | --- | --- | --- | --- | --- | --- | --- | --- | --- | --- |
| **Item** | **Response format** | | | | | | | | | | |
| How well are you coping with the stress of your job right now? | Not well |  |  |  |  |  |  |  |  |  | Very well |
