# Supplementary material for: Examining the impact of a universal social and emotional learning intervention (Passport) on internalising symptoms and other outcomes among children, compared to the usual school curriculum: study protocol for a school-based cluster randomised trial
Source: Trials. 2023 Nov 2;24:703. doi: 10.1186/s13063-023-07688-0 (PMC10621084; doi:10.1186/s13063-023-07688-0)
Supplement: Supplementary file 1 — Additional file 1. Data collection tools. [file 13063_2023_7688_MOESM1_ESM.zip › Additional file 1. /Teacher interview schedule visit 2 version 3.docx]

**Kavli Teacher Interview Schedule visit 2**

**Introduction**

**Preamble**

*Provide participant with copy of information sheet (they will have already received a copy in email), offer a summary of key areas, and confirm that they have read this and understood*

Like last time, our conversation will take about an hour. There are no right or wrong answers, we are just interested in what you think. This evaluation is separate from the Passport intervention team so we don’t have any vested interests in the intervention, and we want you to be honest with us about how you are finding Passport.

We will record our conversation so that we can write down all the things you tell us. We may write about the things that you tell us in reports, but other people outside of your school won’t be able to tell that it’s you. At the end of the project, we will share a written version of the conversations that we have together, but we won’t tell anyone your name and people who see this won’t be able to tell that it’s you. Because we are only visiting a small number of schools, it is possible that colleagues in the school may know that it is you in our report when we write about how teachers and children have experienced delivering Passport. However, we are not asking questions that we think would affect your reputation, and people outside of your school will not be able to tell that it is you.

**Ask**: are you happy to chat to me again today about your experiences of delivering Passport with your class?

**Ask**: do you have any questions before we start?

*Provide consent form and take written consent from the participant before beginning.*

**Ask**: Is it okay for me to start recording now?

**Schedule**

*Various prompts shown in relation to questions. More widely, probe answers to help unpack points, and elicit details and concrete examples.*

**A. Implementation of Passport**

*Looking for information about dosage, fidelity and adaptation, reach and responsiveness, quality, and factors affecting implementation. Can bring through points from previous interview as appropriate.*

1. Can you tell me your overall impression of Passport?
   1. What did you think of the content of the sessions?
   2. What did you think of the design of the materials?
2. Can you tell me about how many of the Passport sessions you delivered?
   1. How have you found it fitting these into the week?
   2. Do you think that Passport should have more or less sessions? If so, can you tell me a little bit more about what you think would be helpful about that kind of change?
3. We know that teachers quite often make changes to social and emotional learning lessons – changing scenarios or wording, leaving some parts out, etcetera. These might be big changes or little changes, and it might be something that’s changed *every* session or it might be about one particular activity one week. Have you made any changes to the sessions when you have been delivering them?
   1. *Follow up to probe their answer – e.g., how often did they do X, to what extent?*
   2. *If yes*: can you walk me through the thinking behind those changes?
   3. *If yes:* How did these changes worked in practice?
   4. *If no:* Were there any changes you thought about making?
4. How do you think your students responded to Passport sessions?
   1. Can you give me any examples of how students have been responding?
   2. How do they respond to the materials?
   3. How do they react to the scenarios?
5. If other teachers were to deliver Passport in their classrooms, do you think there’s anything they should know about how to do it well?
6. Is there anything about your school that you think is helpful or unhelpful when you are trying to incorporate Passport?
7. What do you think are the similarities and differences between Passport and other wellbeing initiatives or practices in which the school has been involved?
8. We understand there is booster training for Passport partway through delivery – is this something you attended?
   1. Can you tell me about anything you found helpful about the booster training?
   2. Can you tell me about anything you found less helpful about the booster training, or anything you think could have been done differently or better?

**B. Impact and helpful aspects of Passport**

*Looking for information about impact for children, including any differential impact*

1. Are there any modules or sessions that you have found particularly well suited to the needs of your class? [Remind that file is available]
   1. You mentioned in our last interview [insert relevant school context or class need as noted in Section A] - how do you think that Passport as a whole relates back to that?
2. Do you think any of the children in your class have benefited from Passport?
   1. In what ways do you think they have benefited?
   2. If unclear: is this the same for all children, or do you think particular children have benefited more or less than one another? Can you tell me about why you think that is?
   3. Can you give me any examples of ways that you think children have benefited?
   4. Do you think you can still see any of those benefits now?
   5. *If no:* can you tell me your thoughts about why Passport may not have benefitted children in your class?
3. We know interventions like Passport often have lots of aspects to them, and some things might be seen as really helpful, almost like “key ingredients” - from your experience of delivering Passport, what do you think the key ingredients are in it?

**C. Sustainability**

*Looking for information about continued use of aspects of Passport, and factors that might contribute to this. Unpack thinking behind answers throughout.*

1. Would you continue to use any of the strategies or materials you have explored in Passport with the children in your current class?
2. Would you use Passport approaches with future class groups?
3. Would you recommend Passport to colleagues in other schools?
   1. *If yes:* How might you suggest they go about embedding this in their school?
   2. *If no:* Can you tell me a little more about this answer?

**Conclusion**

That’s all my questions for you. Is there anything else you would like to add?

*Stop recording now.*

Thank you so much for talking with us, it’s been helpful to talk to you and understanding your experiences of delivering Passport.

To remind you, we’re going to write about the things we’ve been discussing in reports but other people outside of the school won’t be able to tell that it’s you in those reports. We will share a written version of our conversations with other people, but we won’t tell them that this is you.

Do you have any questions for us now that we’ve finished? If you think of any questions later, you can email us again.
