# Supplementary material for: Examining the impact of a universal social and emotional learning intervention (Passport) on internalising symptoms and other outcomes among children, compared to the usual school curriculum: study protocol for a school-based cluster randomised trial
Source: Trials. 2023 Nov 2;24:703. doi: 10.1186/s13063-023-07688-0 (PMC10621084; doi:10.1186/s13063-023-07688-0)
Supplement: Supplementary file 1 — Additional file 1. Data collection tools. [file 13063_2023_7688_MOESM1_ESM.zip › Additional file 1. /v2 Kavli Child Focus Group Guide Visit 1.docx]

# **
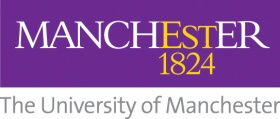
Kavli Child Focus Group Guide 1**

**Introduction**

**Preamble** *(use visual group agreement to point to symbols for each point to help children follow)*

Schools do lots of things to help children feel happy and okay. Some schools have been running a programme called “Passport” and we want to know what that has been like for the children in these schools. We are talking to children in some other schools too, to see what they think.

**Ask:** Did you all read this information sheet that tells you about what you are doing today? *If some say no, read this aloud along with them.*

Are you okay to chat to us about this? This will take about an hour. We will ask you some questions, but if you don’t want to answer then that is okay. This isn’t a test and there aren’t any right or wrong answers – we are just interested in what you all think. You can be honest with us about your thoughts – we didn’t make Passport, and we won’t be upset if there are things you don’t like about it! It’s also okay to disagree with each other, because we know some of you might have different feelings about things, and that’s really helpful for us to understand.

We are going to record our conversation [show Dictaphone], so we can remember and listen again to all the things you tell us.

It’s okay for you to tell other people that we had this conversation today. But because I am asking you what **you** think, it’s important that we don’t tell others what each of you have said, because that isn’t fair. If someone talks about something that makes us think that they are at risk of harm, or someone else is at risk of harm, then we will need to share this with [name of safeguarding contact] so that someone can help.

**Ask**: Do you have any questions?

**Assent**

Are you happy to join in? If not, this is fine! Just let me know. No one will mind.

*(If a pupil decides at this point that he/she does not wish to participate, they can return to the rest of their class)*

*Provide assent form and go through questions together to confirm understanding. Children need to provide written assent and return the form before beginning.*

***Final check***

**Ask**: Do you have any questions before we start?

**Ask**: Is it okay for me to start recording now?

***Reminders throughout discussion…***

*Various prompts shown in relation to questions. More widely, probe answers to help unpack points, elicit details and concrete examples, and explore group consensus or differences. Try to use the children’s language back to them in probing. Examples:*

- *Can you tell me more about X?*
- *Can you give me an example of X?*
- *What makes you think X?*
- *How did you feel about X?d*
- *What do you mean when you say X?*

*Prize answers rather than praising. E.g., instead of praise such as “that’s good!” or “well done”, explain neutrally what is helpful about their answer “it’s really helpful to hear your thoughts on X”, or “you gave me lots of detail then and that’s really helped me understand what you think.”*

**A. Impressions of Passport**

*Introducing the focus and looking for perceptions of the purpose of Passport and overall impressions.*

1. We’re here to talk to you about ‘Passport.’ Do you know what Passport is? *Show some of the materials.*
   1. *If some or all recognise the name, use flipchart paper and felt tips together to map out:* It would be really helpful if you can all tell me a bit more about what Passport is. We can all write or draw what comes to mind when you think about Passport and what you do together in lessons.

*Unpack answers on paper together, asking about their answers and comparing similar and different answers about what happens in Passport*

- 1. Why do you think schools use Passport?
  2. *If some do not know what this refers to still, explain and show basic material examples from package:* Passport is a set of lessons schools can use to help children feel happy and okay. Do you recognise these things from lessons you have been doing about feeling happy and okay?

1. Think back to the first time you did a Passport lesson. How did you feel about it? *Use post-it-notes to have them write down their answers and stick on flipchart paper OR on the feelings poster from Passport, and unpack answers.*
   1. What about now? How do you feel doing the lessons? *Unpack answer, whether same or different.*
   2. How do you think your teacher feels when they teach you Passport lessons? *Use post-it notes again.*

**B. Experiences of engaging with Passport**

*Looking for general experiences of engaging with sessions and materials. Questions are deliberately broad, to use as starting points for ongoing discussion. Prompt throughout to explore for details and concrete examples; use materials if helpful to support discussion (points to explore and unpack could be session content/scenarios, comics and other resources, teacher delivery)*

1. We’d like to know about the things you like and don’t like about Passport. Here, we have a green bucket and a red bucket. I’d like you to write one thing you do like about Passport on a post-it note and put it in the green bucket, and one thing you don’t like about Passport and put it in the red bucket. Then we can get them out and talk together about the things you wrote – you don’t have to put your name on it, and when we get your post-it note out you can tell us if you want to but you don’t have to! Remember, you can be honest about what you do and don’t like, and it’s okay if some of you feel differently about things.
2. What would you change about Passport? *If helpful, point them back to things they said they didn’t like as a starting point, but encourage to think about any other things as well to avoid missing more subtle changes.*

**C. Impact and helpful aspects of Passport**

*Looking for information about initial impact*

1. Are there any things you have learned so far from doing Passport lessons?
   1. *If yes:* Can you give me some examples?
   2. How do you think the lessons helped you learn those things?
   3. Do you ever think about the things you have been learning in Passport in real life situations?
   4. Do you think you’ll use the things you’ve learned from Passport as you grow up?
   5. *Move straight here if they answer no, OR introduce after they have gone through points they have learned:* Are there things you would have liked to learn? Are there ways that Passport could have helped you learn things better?
2. Do you think that Passport is helpful for your class? (Note: steer away if they talk about specific peers, be clear we mean generally. If they find this hard, use “children your age”)
   1. *If yes, unpack, starting with:* Can you tell me what you think is helpful about Passport for your class?
   2. *If no, unpack, starting with:* Can you tell me what makes you think Passport is not helpful for your class? How would you make it helpful?

**Conclusion**

That’s all our questions for you. Is there anything else you would like to add?

Thank you so much for talking with us, it’s been helpful to talk to you and we’ve learned a lot. We’ll be coming back again after you have finished all the Passport lessons, to talk to you again about how you found it all.

To remind you, we will write down the conversations we had today and share them, but we won’t tell people that it was you who said things. We’re also going to write about your ideas in reports, but other people won’t be able to tell that it’s you.

Do you have any questions for us now that we’ve finished? If you think of any questions later, your school or your parent or guardian can help you get in touch with us to ask us.

**Reminder:** provide certificate and sources of support
